# PODN is a prognostic biomarker and correlated with immune infiltrates in osteosarcoma

**DOI:** 10.1186/s12935-021-02086-5

**Published:** 2021-07-17

**Authors:** Feng Yao, Zhao Feng Zhu, Jun Wen, Fu Yong Zhang, Zheng Zhang, Lun Qing Zhu, Guang Hao Su, Quan Wen Yuan, Yun Fang Zhen, Xiao Dong Wang

**Affiliations:** 1 Department of Orthopedics, Children’s Hospital of Soochow University, Su Zhou, 215025 Jiang Su China; 2 Clinical Pediatric School of Soochow University, Su Zhou, 215025 Jiang Su China; 3 Clinical Pediatric Institute, Children’s Hospital of Soochow University, Su Zhou, 215025 Jiang Su China

**Keywords:** Osteosarcoma, PODN, Diagnosis, Immune infiltrates, Prognosis, Biomarker

## Abstract

**Background:**

Osteosarcoma was the most common primary bone malignancy in children and adolescents. It was imperative to identify effective prognostic biomarkers for this cancer. This study was aimed to identify potential crucial genes of osteosarcoma by integrated bioinformatics analysis.

**Methods:**

Identification of differentially expressed genes from public data gene expression profiles (GSE42352), functional and pathway enrichment analysis, protein–protein interaction (PPI) network construction and module analysis, Cox regression and survival analysis was conducted.

**Results:**

Totally 17 co-differential genes were found to be differentially expressed. These genes were enriched in biological processes, Kyoto Encyclopedia of Genes and Genomes (KEGG) and Gene Set Enrichment Analysis (GSEA) pathway of inflammatory immune response. PPI network was constructed with 63 differentially expressed genes that co-existed between the test set and the validation set. The area under the receiver operating characteristic curve (AUC value) was 0.855, which indicated that the expression of PODN had a good diagnostic value for osteosarcoma. Furthermore, Cox regression and survival analysis revealed 5 genes were statistically significant.

**Conclusions:**

PODN was regarded as a potential biomarker for the diagnosis and prognosis of osteosarcoma, ACTA2, COL6A1, FAP, OLFML2B and COL6A3, can be used as potential prognostic indicators for osteosarcoma.

## Introduction

Osteosarcoma was a kind of highly aggressive tumor [1], most often developing in the long bone metaphysis, which have a relatively high incidence in adolescents (0.8–1.1 per 100,000 per year at age 15–19 years) [2, 3]. Compared with surgery alone, multimodal neoadjuvant chemotherapy (ChT) of high-grade localised OS increases disease-free survival probability from 10%–20% to > 60% [4], and no additional treatment have been found that can increase survival significantly [5, 6].

With the development of molecular biology, many studies have shown that high chromosome instability was one of the most important characteristic hallmark of osteosarcoma. Many studies on osteosarcoma find that mRNA play a potential biological role in osteosarcoma and can be used as prognostic markers and therapeutic targets. However, the biological function and related network of mRNA in osteosarcoma has not been fully elucidated. For instance, PODN gene encodes protein podocan. Podocan was a small proteoglycan (SLRP) family protein rich in leucine. It was not only a strong regulator of cell phenotype in extracellular matrix (ECM), but also a kind of ECM protein. ECM protein was involved in cell migration and proliferation, and high podocan level inhibits the proliferation of (SMC) in smooth muscle cells [7], so PODN may also be involved in the regulation of cell proliferation. PODN is associated with malignant tumors, YiBai et al. established a prognostic model of gastric cancer (GC) through PODN and other 6 genes, which can accurately predict the prognosis of GC [8].

In this study, bioinformatics methods were used to integrate the mRNA expression data, which was available in the GEO database, to identify differentially expressed mRNA between osteosarcoma and normal cell, and establish the related network of targeted molecules, aiming to provide new targets for diagnosis, treatments, and prognostic in osteosarcoma. Finally, it was determined that PODN has functional relevance in osteosarcoma and additional genes (ACTA2, COL6A1, FAP, OLFML2B and COL6A3) have prognostic significance for osteosarcoma.

## Materials and methods

### Identification of differential (co-expressed) genes from public microarray data

It was found that the expression of PODN had a significant difference between 15 normal samples and 103 osteosarcoma samples in the GSE42352 data set (test set), suggesting that PODN could be used as a diagnostic and prognostic biomarker for osteosarcoma. Then, 103 osteosarcoma samples in GSE42352 data set were grouped according to the median expression of PODN, including 51 cases in the low expression group and 52 cases in the high expression group. Difference analysis was performed by using the limma package [9], 103 differential genes was obtained by screen differential (co-expressed) genes according to multiple of fold change and *P* value (|logFC| > 1 & *P* < 0.05). Pheatmap package and ggplot2 package were used for visualization, heat map and volcano map of differential genes were performed. 101 osteosarcoma samples from the Target-OS data set (validation set) were grouped by the median expression of PODN, including 50 in the low expression group and 51 in the high expression group, and then, 526 differential genes were obtained in the same way. The intersection of two differential genes sets was obtained with 63 co-differential genes (|logFC| > 1 & *P* < 0.05). Thirty-eight differential lncRNAs were also obtained in the Target-OS data set (validation set) (|logFC| > 1 & *P* < 0.05).

### GO, KEGG, and GSEA enrichment analysis

GO (Gene Ontology) includes BP (Biological Process), MF (Molecular Function) and CC (Cellular Component). GO analysis of differential genes (screening criteria was FDR < 0.1) was performed with the clusterProfiler package [10], using ggplot2 (https://cran.r-project.org/web/packages/ggplot2/index.html), R package for visualization. The ClueGO plugin of cytoscape software supports more than 200 species, database real-time update,multiple annotation data sets, multiset enrichment result comparison, and network graph presentation. The plug-in was also used for validation enrichment analysis, and the results were basically consistent. Kyoto Encyclopedia of Genes and Genomes (KEGG) enrichment analysis used the clusterProfiler package and plotted the associated bar graph, bubble graph and enrichment graph. KEGG pathway enrichment analysis was performed on differentially expressed genes (DEGs) with EnsembleID among the differential genes in the test set to obtain an enrichment list, and pathways with *P* < 0.05 were considered significantly enriched.

Gene Set Enrichment Analysis (GSEA) [11] used the expression matrix of differential genes grouped by high and low expression of PODN, which was analyzed by the ssGSEA package, and the selected reference gene set was msigdb.v7.0.entrez.gmt. and the ggplot2 package was used to visualize the GSEA collection.

### Construction of PPI molecular interaction network and extraction of key gene modules

STRING database [12] was used to construct protein-protein interaction networks for differential genes. Currently, the database contains 18,838 human proteins and 25,914,693 core network interactions. In the present study, a protein–protein interaction (PPI) network was constructed by using the DEGs identified by the serial interaction body. The highest confidence interaction score was set to 0.4. The Hub genes of PPI network modules were screened by using MCODE and the cytoHubba plug-in and visualized.

### Difference of immune infiltrating cells and correlation analysis of immunophenotype

CIBERSORT [13] is an algorithm widely used to characterize the cellular composition of complex tissues by gene expression levels in solid tumors. When using CIBERSORT, the LM22 signature algorithm was employed. LM22 is a special gene marker containing 547 genes that can differentiate 22 immune cell hypotypes downloaded from the CIBERSORT portal website (http://cibersort.stanford.edu/). In this study, we used the CIBERSORT package and calculated the infiltrating abundance of 22 immune cell with high and low PODN expression in 103 osteosarcoma samples by the LM22 algorithm, including different T cells, B cells, plasma cells, natural killer cells, and different myeloid lineages. Subsequently, we also analyzed the expression distribution correlation of 22 infiltrating immune cells at high and low PODN expression, and plotted the correlation heat map, with red representing positive correlation and blue representing negative correlation, and the darker the color, the closer the value is to 1 and the stronger the correlation.

From the ImmPort database (https://immport.niaid.nih.gov/home) [14], a list of immune-related genes was obtained. Then, the intersection of the differential genes was acquried in the test set and the validation set with the immunophenotype-related genes and list genes respectively, and a Wayne diagram was made by the VennDiagram package. Subsequently, the pheatmap package of R language was used to correlate the differential gene sets of PODN high and low expression groups with immunophenotype-related genes, and the results with strong correlation were visualized to draw a correlation heat map.

### Area under the ROC curve (AUC) to assess the diagnostic performance

The receiver operating characteristic curve (ROC curve) was plotted by pROC package, with the true positive rate (sensitivity) as the ordinate and the false positive rate (1-specificity) as the abscissa. It is used to analyze whether the expression level of PODN can well distinguish between 15 normal samples and 103 osteosarcoma samples, and determine to produce the optimal cut-off value of the highest likelihood ratio to decide the recognition threshold of PODN for osteosarcoma. The survivalROC package was also used to validate the accuracy of the risk model in predicting the survival prognosis of osteosarcoma. An area under the curve (AUC) greater than 0.5 can visually reflect the diagnostic value of biomarkers for the disease. At the same time, the closer the area under the ROC curve is to 1 and the closer to the (0, 1) point, the better the authenticity of the diagnostic test is demonstrated.

### Cox regression and hazard model construction

At first, univariate Cox regression analysis of the Target database gene matrix was performed. Then, univariate regression analysis results was extracted by a risk factor collection with 17 co-differential genes, and further multivariate Cox regression analysis (narrow the range) of the gene collection was performed for predicting high-risk biomarkers for osteosarcoma.

### Survival analysis

Target-OS data set survival analysis was performed using the survival package data, including overall survival time (OS), event-free (disease) survival time (DFS), and time to progression (TTP). To validating if the risk model has a good accuracy for the overall survival prognosis of osteosarcoma.

### Statistical analysis

Excel (Microsoft) and R statistical software (version 4.0.2) were used to analyze data. *P* value less than 0.05 was considered statistically significant.

## Results

### Differential expression analysis

**Fig. 1:**
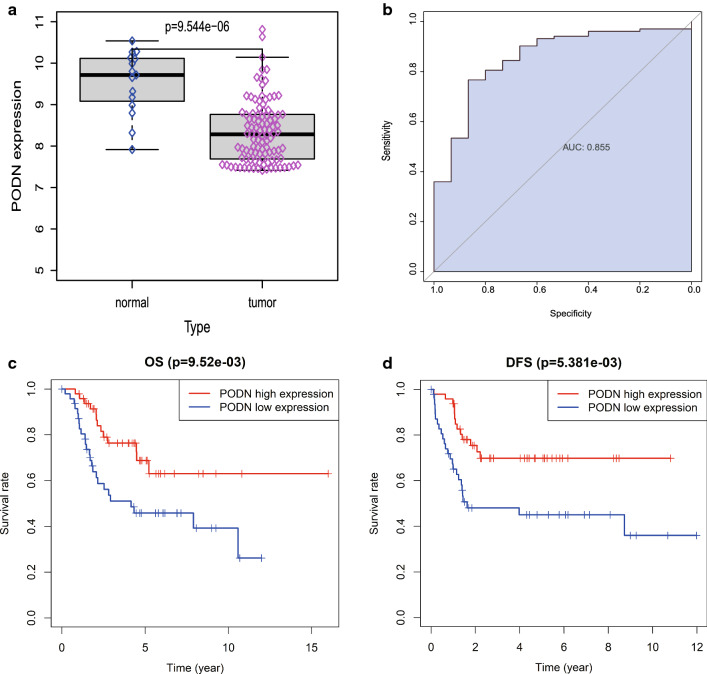
Differential expression and prognostic significance of PODN in osteosarcoma. **A** PODN was significantly highly expressed in tumor tissues compared with normal tissues; **B** PODN was used to assess the diagnostic performance of PODN for osteosarcoma by area under the ROC curve (AUC) in the test set; **C** the effect of PODN on the overall survival prognosis (OS) of osteosarcoma was statistically significant; **D** the effect of PODN on the progression-free survival prognosis (DFS) of osteosarcoma disease was statistically significant

**Fig. 2:**
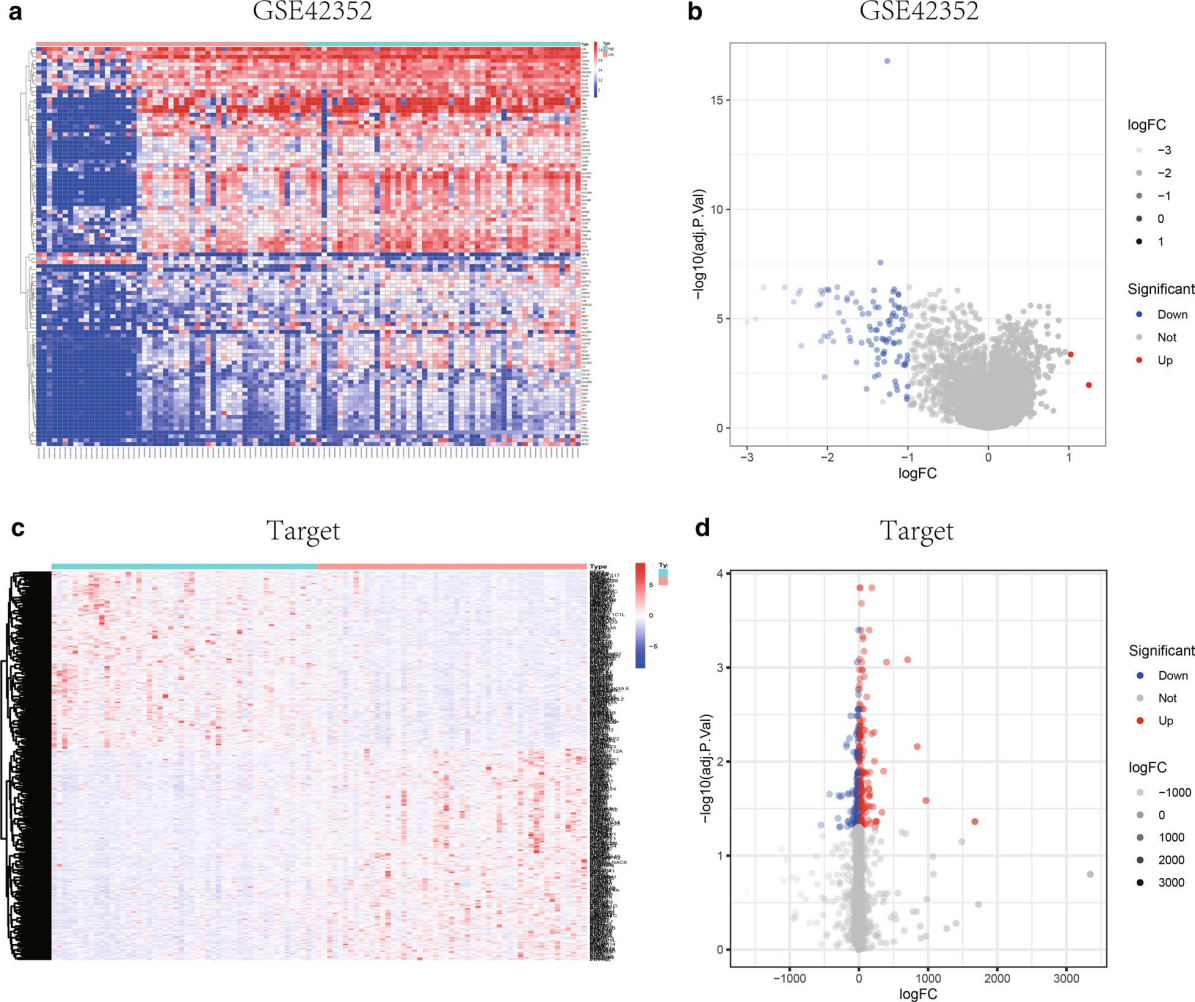
Differential analysis of single and co-expressed genes. **A** The median expression levels of PODN in 103 osteosarcoma samples of GSE42352 data set (test set) were divided into high and low expression groups, and the significant differential genes between the two groups were displayed in the form of heat map; **B** the significant differential genes between the two groups of GSE42352 data set were displayed in the form of volcano diagram; **C** the median expression levels of PODN in 101 osteosarcoma samples of Target-OS data set were divided into high and low expression groups, and the significant differential genes between the two groups were displayed in the form of heat map; **D** the significant differential genes between the two groups of Target-OS data set were displayed in the form of volcano diagram

The GSE42352 data set of 15 normal samples and 103 osteosarcoma samples was used as the test set for differential analysis. Then, we found that PODN was significantly different between normal samples and osteosarcoma patients (Fig. 1A). Subsequently, we grouped the 103 tumor samples of the test set into high and low expression groups by PODN median value, including 51 cases in the low expression group and 52 cases in the high expression group. Differential analysis was performed by using the limma package and the differential results were visualized in the form of volcano maps and heat maps (Fig. 2A, B). And 101 osteosarcoma patients in the Target database of the validation set were divided into high and low expression groups by the median value of PODN expression, 50 and 51 cases respectively. Differential analysis was performed using the limma package, and differential (co-expressed) genes were screened according to multiple of difference between groups (fold change) and significance of difference (*P* value), setting a threshold of |logFC| > 1 & *P* < 0.05, to obtain 103 co-expressed genes. Pheatmap and ggplot2 packages were also used for visualization to draw the heat map and volcano map of differential genes. The median expression levels were grouped in 101 osteosarcoma samples from the Target-OS dataset (validation set), including 50 in the low expression group and 51 in the high expression group, and 526 differential genes were obtained in the same way (Fig. 2C, D). The intersection of two differential genes sets was obtained with 17 co-differential genes (Fig. 8A).

### GO, KEGG, and GSEA enrichment analysis

**Fig. 3:**
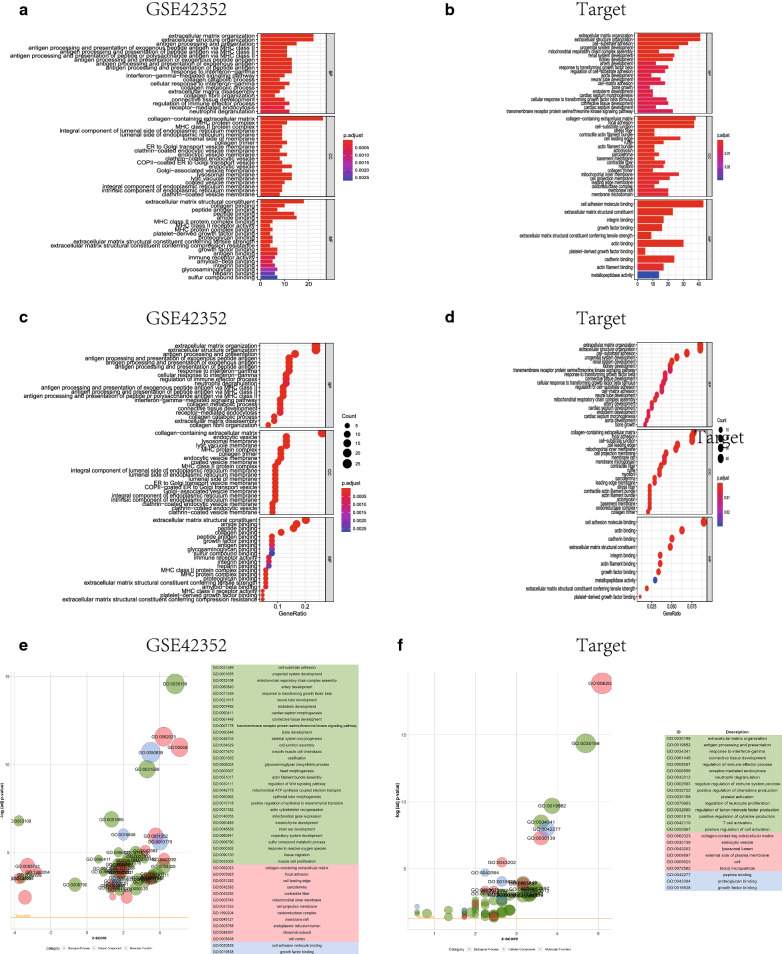
GO enrichment analysis of differential genes. GO enrichment analysis was performed for GSE42352 and Target database differential genes respectively. **A** The bar graph of GO enrichment analysis of GSE42352 data set using clusterProfiler package. Length represents the number of gene enrichment, color represents significance; **B** the bar graph of GO enrichment analysis of Target-OS data set using clusterProfiler package. Length represents the number of gene enrichment, color represents significance; **C** the bubble graph of GO enrichment analysis of GSE42352 data set using clusterProfiler package. Bubble size represents the number of gene enrichment, color represents significance; **D** the bubble graph of GO enrichment analysis of Target-OS data set using clusterProfiler package. Bubble size represents the number of gene enrichment, color represents significance; **E** the GO enrichment results of using GoPlot package to analyse test set were used to draw bubble diagram; **F** the GO enrichment results of using GoPlot package to analyse validation set were used to draw bubble diagram

**Fig. 4:**
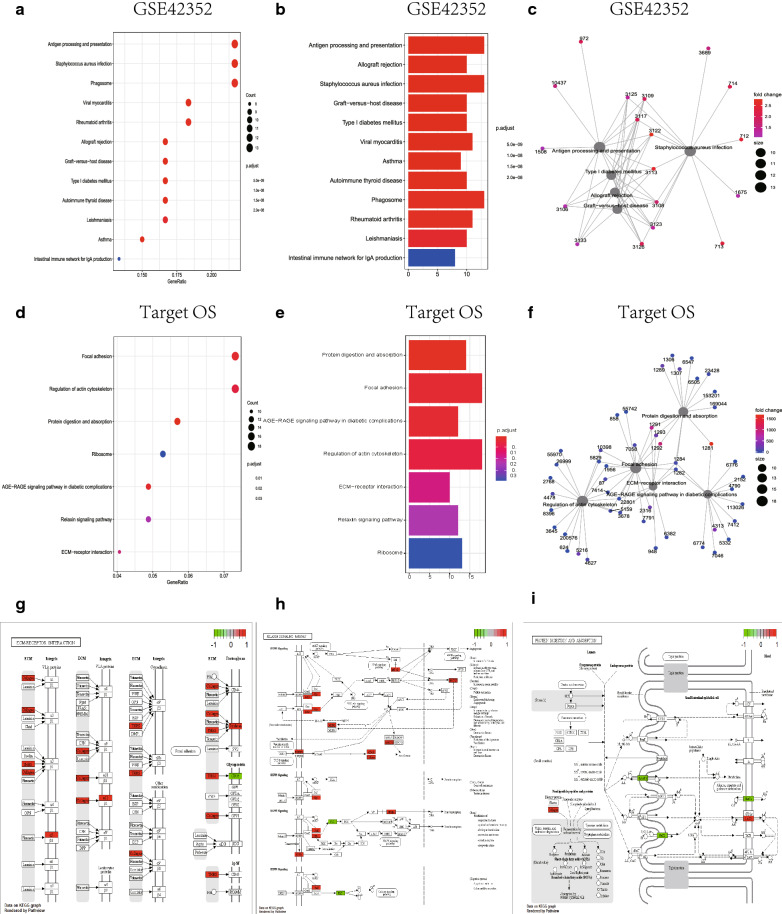
KEGG enrichment analysis. KEGG enrichment analysis was performed for GSE42352 and Target database differential genes, respectively. **A** The bubble graph of KEGG enrichment analysis of GSE42352 data set using clusterProfiler package. Bubble size represents the number of gene enrichment, color from blue to red, significance gradually increased; **B** the bar graph of KEGG enrichment analysis of GSE42352 data set using clusterProfiler package. Length represents the number of gene enrichment, color from blue to red, significance gradually increased; **C** the enrichment diagram of KEGG analysis of Target-42352 data set using clusterProfiler package, showing the relationship between gene ID and pathway; **D** the bubble graph of KEGG enrichment analysis of Proteet-OS data set using clusterProfiler package. Bubble size represents the number of gene enrichment, color from blue to red, significance gradually increased; **E** the bar graph of KEGG enrichment analysis of Target-OS data set using clusterProfiler package. Length represents the number of gene enrichment, color from blue to red, significance gradually increased; **F** the enrichment diagram of KEGG analysis of Target-OS data set using clusterProfiler package, showing the relationship between gene ID and pathway; **G** ECM-receptor interaction pathway; **H** relaxin signaling pathway pathway; **I** protein digestion and absorption pathway

GO (Gene Ontology) includes BP (Biological Process), MF (Molecular Function) and CC (Cellular Component). GO analysis of differential genes (Fig. 3 and Table 1) was performed with the clusterProfiler package [10] (screening criteria was *FDR* < 0.1) and visualized using the ggplot2 package. KEGG enrichment analysis (Fig. 4 and Table 2) was performed using the clusterProfiler package and plotting the correlation bar graph, bubble graph, enrichment graph. KEGG pathway enrichment analysis was performed on DEGs with EnsembleID among the differential genes in the test set to obtain an enrichment list, and pathways with *P* < 0.05 were considered significantly enriched.

**Table 1:** List of GO enrichment analysis performed on GSE42352 (gene enrichment number Top10) and target (gene enrichment number Top10) database co-expressed differential genes by clusterProfiler package

Dataset	Ontology	ID	Description	*P*.adjust	Count
GSE42352	BP	GO:0030198	Extracellular matrix organization	4.25E−15	22
GSE42352	BP	GO:0043062	Extracellular structure organization	4.25E−15	22
GSE42352	BP	GO:0019882	Antigen processing and presentation	1.98E−10	15
GSE42352	BP	GO:0002478	Antigen processing and presentation of exogenous peptide antigen	8.65E−10	13
GSE42352	BP	GO:0019884	Antigen processing and presentation of exogenous antigen	1.25E−09	13
GSE42352	BP	GO:0048002	Antigen processing and presentation of peptide antigen	1.79E−09	13
GSE42352	BP	GO:0034341	Response to interferon-gamma	3.09E−09	13
GSE42352	BP	GO:0071346	Cellular response to interferon-gamma	1.19E−08	12
GSE42352	BP	GO:0002697	Regulation of immune effector process	0.000287702	12
GSE42352	BP	GO:0043312	Neutrophil degranulation	0.00042594	12
GSE42352	CC	GO:0062023	Collagen-containing extracellular matrix	1.39E−19	26
GSE42352	CC	GO:0010008	Endosome membrane	9.35E−07	14
GSE42352	CC	GO:0030139	Endocytic vesicle	4.96E−08	13
GSE42352	CC	GO:0005765	Lysosomal membrane	2.61E−07	13
GSE42352	CC	GO:0098852	Lytic vacuole membrane	2.61E−07	13
GSE42352	CC	GO:0005774	Vacuolar membrane	1.09E−06	13
GSE42352	CC	GO:0042611	MHC protein complex	1.00E−17	11
GSE42352	CC	GO:0005581	Collagen trimer	1.39E−11	11
GSE42352	CC	GO:0030666	Endocytic vesicle membrane	1.26E−08	11
GSE42352	CC	GO:0030133	Transport vesicle	2.34E−05	11
GSE42352	MF	GO:0005201	Extracellular matrix structural constituent	3.04E−17	18
GSE42352	MF	GO:0033218	Amide binding	1.06E−08	15
GSE42352	MF	GO:0042277	Peptide binding	1.06E−08	14
GSE42352	MF	GO:0005518	Collagen binding	1.21E−10	10
GSE42352	MF	GO:0004175	Endopeptidase activity	0.010282087	8
GSE42352	MF	GO:0042605	Peptide antigen binding	1.02E−08	7
GSE42352	MF	GO:0019838	Growth factor binding	8.98E−05	7
GSE42352	MF	GO:0003823	Antigen binding	0.000230162	7
GSE42352	MF	GO:0005539	Glycosaminoglycan binding	0.001734373	7
GSE42352	MF	GO:1901681	Sulfur compound binding	0.002670804	7
Target-OS	BP	GO:0030198	Extracellular matrix organization	6.15E−12	41
Target-OS	BP	GO:0043062	Extracellular structure organization	6.15E−12	41
Target-OS	BP	GO:0031589	Cell-substrate adhesion	2.67E−07	33
Target-OS	BP	GO:0001655	Urogenital system development	0.000137365	27
Target-OS	BP	GO:0034329	Cell junction assembly	0.010818905	25
Target-OS	BP	GO:0072001	Renal system development	0.00046375	24
Target-OS	BP	GO:0001503	Ossification	0.014822976	24
Target-OS	BP	GO:0001822	Kidney development	0.00057211	23
Target-OS	BP	GO:0007178	Transmembrane receptor protein serine/threonine kinase signaling pathway	0.00844363	23
Target-OS	BP	GO:0030111	Regulation of Wnt signaling pathway	0.016578746	22
Target-OS	CC	GO:0062023	Collagen-containing extracellular matrix	1.28E−09	38
Target-OS	CC	GO:0005925	Focal adhesion	2.57E−09	37
Target-OS	CC	GO:0030055	Cell-substrate junction	2.83E−09	37
Target-OS	CC	GO:0031252	Cell leading edge	9.36E−05	28
TargetOS	CC	GO:0005743	Mitochondrial inner membrane	0.001986238	27
Target-OS	CC	GO:0005759	Mitochondrial matrix	0.043195064	22
Target-OS	CC	GO:0031253	Cell projection membrane	0.002293285	21
Target-OS	CC	GO:0045121	Membrane raft	0.003431572	20
Target-OS	CC	GO:0098857	Membrane microdomain	0.003431572	20
Target-OS	CC	GO:0098589	Membrane region	0.005357409	20
Target-OS	MF	GO:0050839	Cell adhesion molecule binding	1.46E−08	43
Target-OS	MF	GO:0005201	Extracellular matrix structural constituent	3.19E−08	23
Target-OS	MF	GO:0005178	Integrin binding	2.43E−05	17
Target-OS	MF	GO:0019838	Growth factor binding	0.00016495	16
Target-OS	MF	GO:0030020	Extracellular matrix structural constituent conferring tensile strength	0.00016495	9
Target-OS	MF	GO:0003779	Actin binding	0.000286309	30
Target-OS	MF	GO:0048407	Platelet-derived growth factor binding	0.000577035	5
Target-OS	MF	GO:0045296	Cadherin binding	0.001118768	24
Target-OS	MF	GO:0051015	Actin filament binding	0.002243868	17
Target-OS	MF	GO:0008237	Metallopeptidase activity	0.029742453	14

**Table 2:** List of KEGG enrichment analysis performed on GSE42352 (gene enrichment number Top10) and target (gene enrichment number Top10) database co-expressed differential genes by clusterProfiler package

Dataset	ID	Description	*P*	qvalue	Count
Target-OS	hsa04974	Protein digestion and absorption	0.0007	0.0006	14
Target-OS	hsa04510	Focal adhesion	0.0042	0.0037	18
Target-OS	hsa04933	AGE–RAGE signaling pathway in diabetic complications	0.0042	0.0037	12
Target-OS	hsa04810	Regulation of actin cytoskeleton	0.0074	0.0065	18
Target-OS	hsa04512	ECM-receptor interaction	0.0170	0.0150	10
Target-OS	hsa04926	Relaxin signaling pathway	0.0240	0.0211	12
Target-OS	hsa03010	Ribosome	0.0392	0.0345	13
GSE42352	hsa04612	Antigen processing and presentation	0.0000	0.0000	13
GSE42353	hsa05150	*Staphylococcus aureus* infection	0.0000	0.0000	13
GSE42354	hsa04145	Phagosome	0.0000	0.0000	13
GSE42355	hsa05152	Tuberculosis	0.0000	0.0000	12
GSE42356	hsa05416	Viral myocarditis	0.0000	0.0000	11
GSE42357	hsa05323	Rheumatoid arthritis	0.0000	0.0000	11
GSE42358	hsa05322	Systemic lupus erythematosus	0.0000	0.0000	11
GSE42359	hsa04514	Cell adhesion molecules	0.0000	0.0000	11
GSE42360	hsa05166	Human T-cell leukemia virus 1 infection	0.0000	0.0000	11
GSE42361	hsa05168	Herpes simplex virus 1 infection	0.0039	0.0026	11

**Fig. 5:**
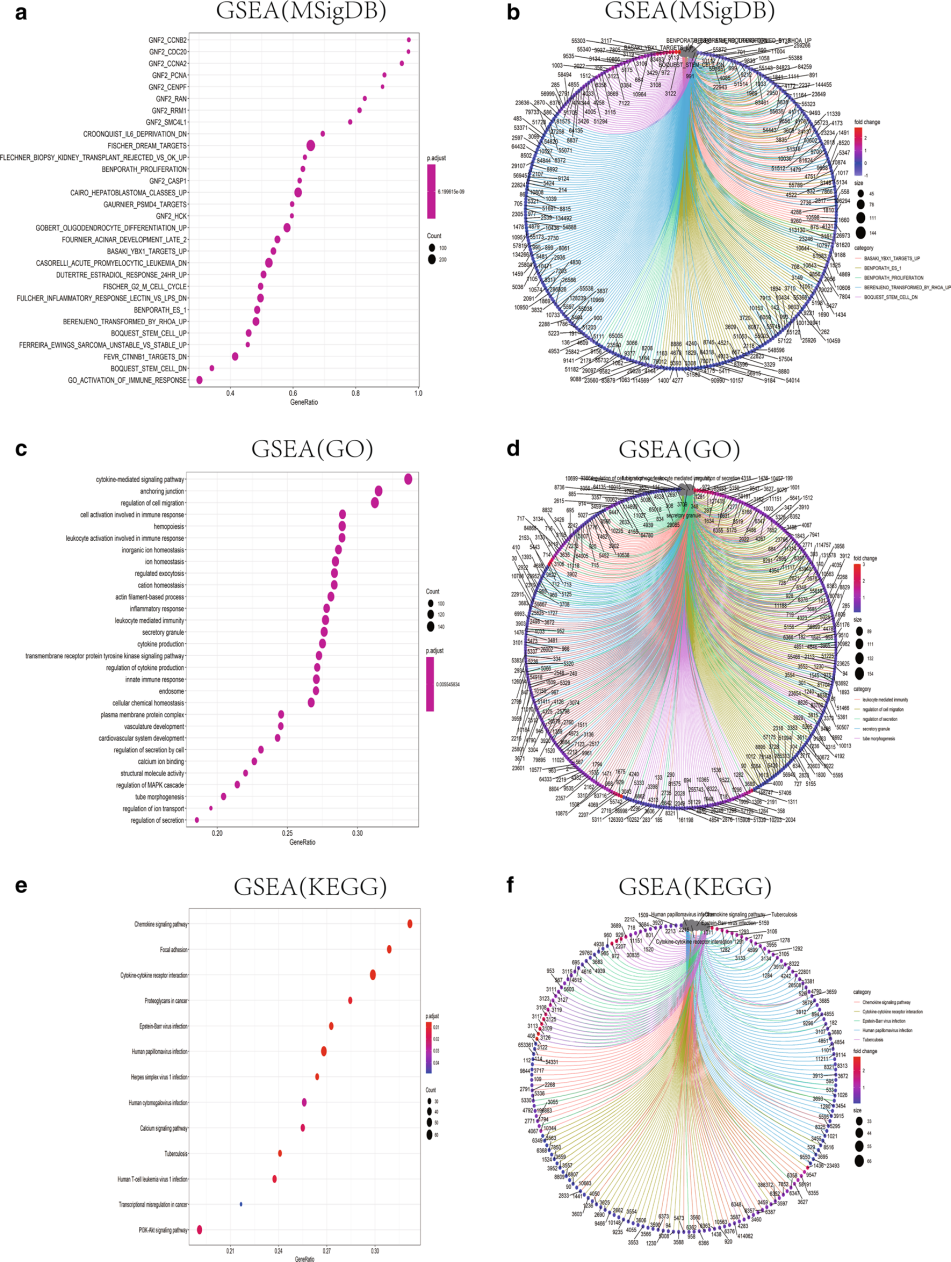
GSEA enrichment analysis. GSEA enrichment analysis was performed using the ssGSEA package on the GSE42352 database with the results of PODN high and low expression group difference analysis. **A**, **B** The forms of bubble graph (**A**) and enrichment chord graph (**B**) based on GSEA enrichment analysis of MSigDB database show the Top30 enrichment results obtained using ssGSEA method respectively; **C**, **D** The forms of bubble graph (**C**) and enrichment chord graph (**D**) based on GO functional enrichment analysis show the Top30 enrichment results obtained using ssGSEA method respectively; **E**, **F** the forms of bubble graph (**E**) and enrichment chord graph (**F**) based on KEGG database GSEA enrichment analysis show the Top30 enrichment results obtained using ssGSEA method respectively

GSEA enrichment (Fig. 5) analysis used the expression matrix of differential genes grouped by high and low expression of PODN, which was analyzed by the ssGSEA package, and the selected reference gene set was msigdb.v7.0.entrez.gmt. And the GSEA set was visualized. This suggests that high PODN expression may be associated with the above enrichment pathways and molecular enrichment function, and may mediate the occurrence of cancer by indirect mechanisms that affect a variety of cytokines and metabolic enzymes of the above pathways or phenotypes.

### PPI network construction and Hub gene screening

**Fig. 6:**
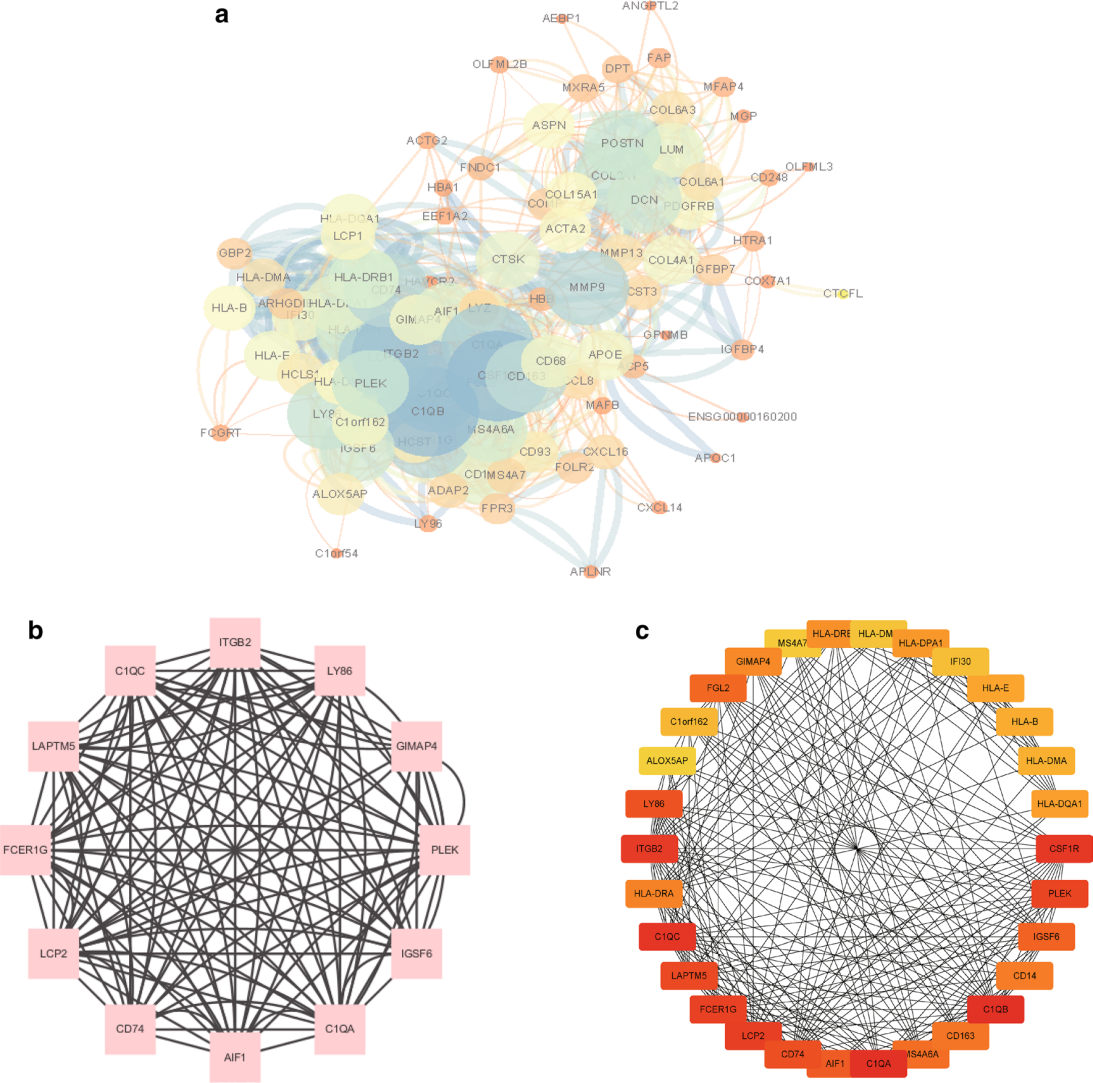
Construction of PPI molecular interaction network. **A** PPI networks were constructed using Cytoscape software on the molecular interaction relationships analyzed by the String database; **B** the most closely related key gene sets obtained through the MCODE plug-in of Cytoscape software; **C** the most closely related Hub gene set with Top30 obtained by the MCC algorithm in the cytoHubba plug-in of Cytoscape software

Subsequently, a protein-protein interaction (PPI) network of differential genes using the STRING database for the 63 differential genes was constructed that co-existed between the test set and the validation set (Fig. 6A). Based on the protein interaction relationship predicted by the string database, one of the most closely linked gene sets was obtained using the MCODE plug-in analysis in Cytoscape (Fig. 6B), as well as the MCC analysis method in the cytoHubba plug-in resulting in a bar diagram of the Hub gene with tight Top30 relationship (Fig. 6C).

### Difference of immune infiltrating cells and correlation analysis of immunophenotype

**Fig. 7:**
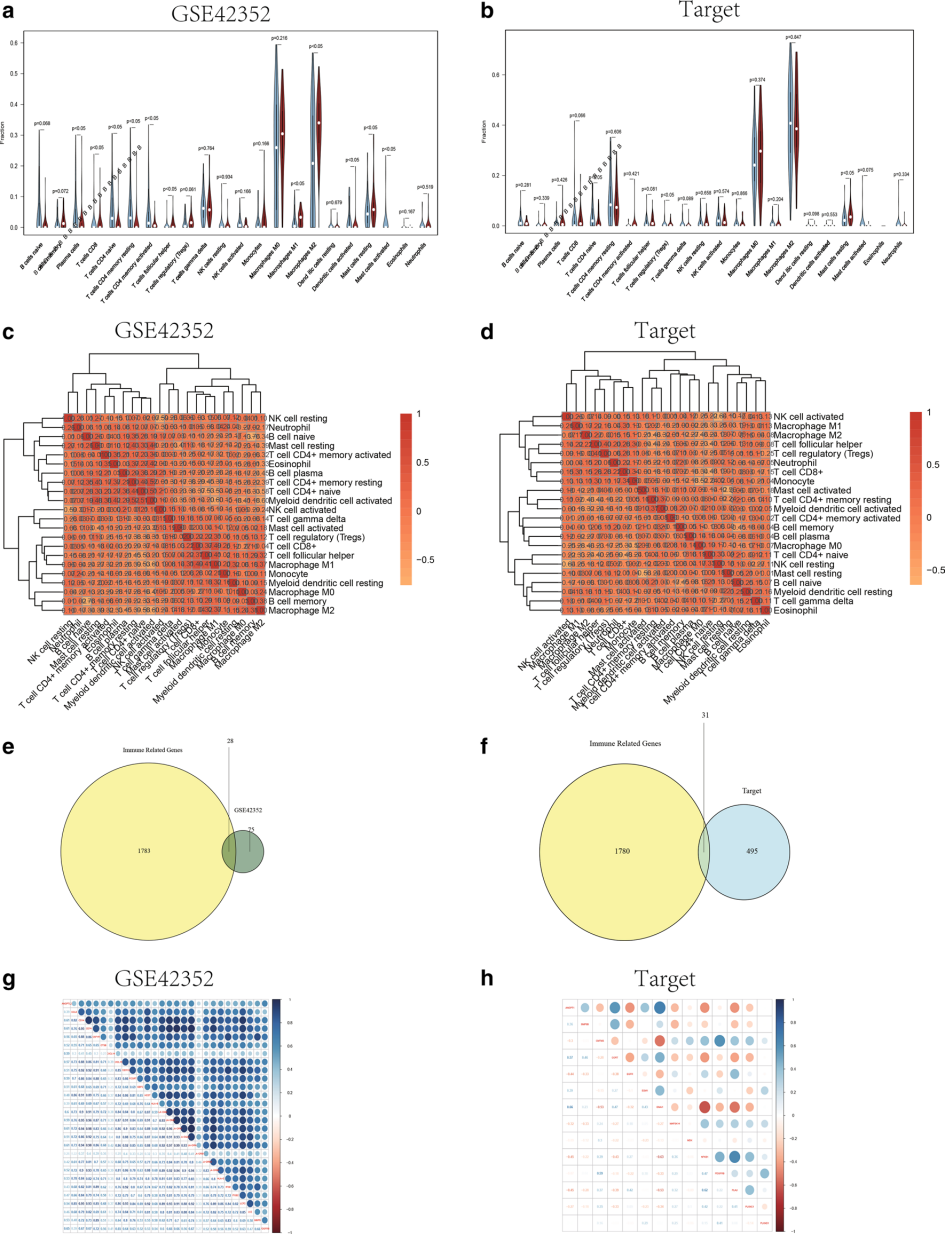
Differential immune cell infiltration and immunophenotype correlation analysis. **A** Using CIBERSORT package, the violin plot of 22 immune cell infiltration expression differences between PODN high and low expression in 103 osteosarcoma samples in the test set was calculated by LM22 algorithm, yellow represents the low expression group and red represents the high expression group, the p value represents the difference significance, and p < 0.05 was considered to have significant difference. **B** The violin plot of 22 immune cell infiltration expression differences between high and low PODN expression in 101 osteosarcoma samples in the validation set, yellow represents the low expression group and red represents the high expression group, p value represents the difference significance, and p < 0.05 is considered to have a significant difference. **C** Heat map of correlation of 22 immune cells between high and low expression of PODN in the test set; **D** heat map of correlation of 22 immune cells between high and low PODN expression set in the validation set; **E** Wayne diagram of intersection of DEGs and immune-related genes in the test set; **F** Wayne diagram of intersection of DEGs and immune-related genes in the validation set; **G** heat map of correlation of immune-related differential gene expression in the test set; **H** heat map of correlation of immune-related differential gene expression in the validation set

Infiltrating immune cell analysis was performed to calculate the infiltrating abundance of 22 immune cell with high and low PODN expression in 103 osteosarcoma samples, including different T cells, B cells, plasma cells, natural killer cells and different myeloid lineages, by using the CIBERSORT inclusion and LM22 algorithms. Subsequently, the differential infiltration expression of 22 infiltrating immune cells at high and low PODN expression was analyzed in the test set and validation set respectively, and plotted a violin plot to show the differential expression of each immune cell between groups (Fig. 7A, B) as well as the correlation heat map (Fig. 7C, D). Combined with the above analysis results, it was suggested that the immune cells with significant differences obtained by us in the high and low PODN expression groups may have a very important role in the immune microenvironment of osteosarcoma. Similarly, the occurrence and development of osteosarcoma may be related to inflammatory and metabolic pathways, and may improve the proportion of immune cell distribution by predicting the target small molecule drugs of these immune cells, so as to achieve the purpose of treating osteosarcoma.

Subsequently, the list of immune-related genes was obtained from the ImmPort database, took the intersection between differential genes and immune-related genes among the test set and validation set, and made a Wayne diagram using the VennDiagram package (Fig. 7E, F). Subsequently, the expression of these differential phenotype-related genes in two data sets was correlated, visualizing the results with strong correlation using pheatmap of R language, and displayed the correlation coefficient to draw the correlation heat map (Fig. 7G, H).

### Diagnostic performance evaluation and survival analysis of PODN molecule for osteosarcoma

Finally, to assess the diagnostic performance of PODN for cancer, receiver operating characteristic curve (ROC curve) was plotted by pROC package for analyzing whether the expression level of PODN could well distinguish 15 normal samples from 103 tumor samples in the test set, and determine to produce the optimal cut-off value of the highest likelihood ratio to decide the recognition threshold of PODN for osteosarcoma. The area under the ROC curve (AUC value) was 0.855, indicating that the expression of PODN has a good diagnostic value for osteosarcoma (Fig. 1B).

In order to further analyze the effect on the survival prognosis of osteosarcoma, the survival package was used to perform the prognostic survival analysis on the clinical overall survival time (OS), event-free (disease) survival time (DFS), and time to progression (TTP) of the validation set, and the results showed that OS and DFS were significantly different (Fig. 1C, D).

### Cox regression analysis and risk score

We first did univariate Cox regression analysis of the Target database gene matrix, and we used 17 common immune-related differential genes as a risk factor set, extracted their univariate regression analysis results, and did further multivariate Cox regression analysis of this gene set. Finally, ACTA2, COL6A1, FAP, OLFML2B and COL6A3 were identified statistically significant (Table 3), indicating that these five differential genes were significantly correlated, and the effect of differential gene set on the prognosis and survival of osteosarcoma was significant, which could be used as the high-risk biomarkers for osteosarcoma.

**Table 3:** The hazard model obtained by multivariate Cox regression analysis based on 17 co-expressed differential genes common to GSE42352 and Target databases

id	coef	exp(coef)	se(coef)	z	Pr( >|z|)
ACTA2	− 0.3465	0.7072	0.1316	− 2.6334	0.0085
COL6A1	− 0.3090	0.7342	0.2067	− 1.4954	0.1348
FAP	− 0.4188	0.6578	0.1896	− 2.2086	0.0272
OLFML2B	− 0.2107	0.8101	0.1367	− 1.5411	0.1233
COL6A3	0.7609	2.1402	0.2538	2.9980	0.0027

**Fig. 8:**
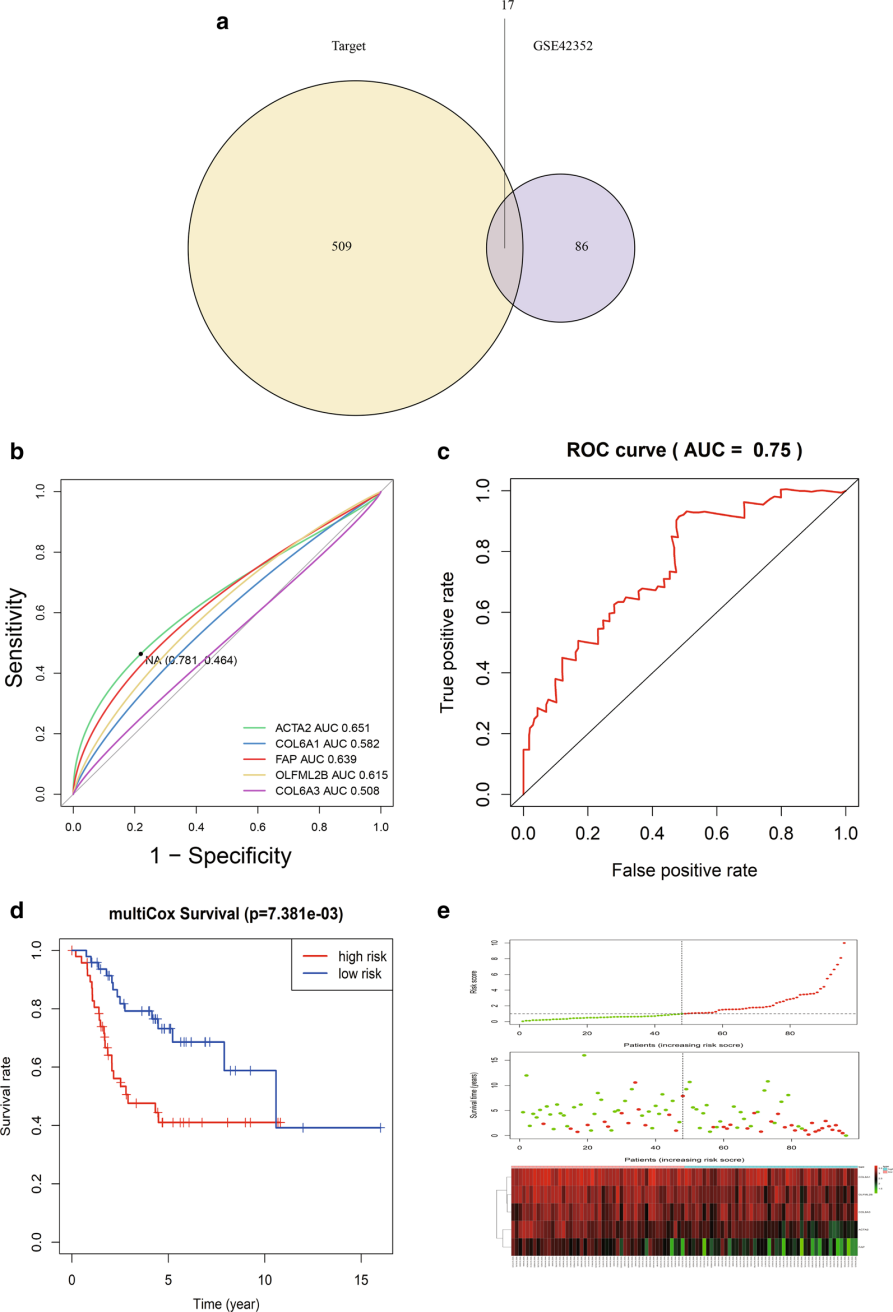
Cox regression analysis and risk assessment analysis. **A** Wayne diagram of the intersection of test set and validation set differential genes; **B** Accuracy of each gene in the 5-gene set (ACTA2, COL6A1, FAP, OLFML2B, COL6A3) obtained by multivariate Cox analysis in predicting the survival prognosis of osteosarcoma; **C** ROC curve verifies the accuracy of the 5-gene set model as a whole in predicting the survival prognosis of osteosarcoma; **D** the effect of the 5-gene set as a risk model on the overall survival (OS) prognosis of osteosarcoma was statistically significant; **E** the risk curve (upper), survival status (middle), and risk heat map (lower) of the 5-gene set

In order to further investigate the effect of the 5 gene set on the survival prognosis of osteosarcoma, we analyzed the overall survival prognosis (OS) (Fig. 8D) between high and low risk groups with it as a risk factor in the clinical survival data of Target database using the survival package. Meanwhile, we used survival ROC package to verify the accuracy of the gene set model in predicting the prognosis of osteosarcoma. We got the result of AUC = 0.75 (Fig. 8C), which indicated that this gene set has high accuracy for the overall survival prognosis of osteosarcoma. Finally, we plotted the risk curve (top), survival status (middle), and risk heatmap (bottom) for this set of gene sets (Fig. 8E).

## Discussion

We designed this experiment to analyze the huge credit generating data and found that the corresponding credit generating indexes after grouping according to different levels of PODN were marked with significantly differences, so we found the corresponding credit generating indexes after grouping according to different levels of PODN. Through bioinformatics analysis (identification of differentially expressed genes from public microarray data, functional and pathway enrichment analysis, PPI network construction and module analysis, Cox regression analysis), we found that the expression of ACTA2, COL6A1, FAP, OLFML2B and COL6A3 genes were significantly different with different PODN expression levels. Further survival analysis showed that they had good accuracy for the overall survival and prognosis of osteosarcoma. Better knowledge on cellular signaling in high-grade osteosarcoma may identify new possibilities for targeted treatment of this cancer [4]. As high-risk biomarkers of osteosarcoma, these five molecules were expected to become potential therapeutic targets, early diagnosis and prognostic indicators.

PODN had an important role of profound clinical significance. For example, studies by YiSun et al. have shown that miR-3180-5p reduces the expression of PODN and leads to cyclin-dependent kinase 2 (cdk2)-mediated proliferation of HBSMCs in human bladder smooth muscle cells [15]. Podocan encoded by PODN can be found in a variety of cells. Ross, Bruggeman et al. find podocan in glomerular basement membrane. And, they elucidate that the imbalance of podocan expression in (HIVAN) of HIV-a-associated nephropathy may lead to the pathogenesis of (FSGS) in focal segmental glomerulosclerosis [16]. Didangelos et al. confirm the existence of Podocan in aortic intima by immunohistochemical staining [17]. Many related researches were carired to verify the importance of PODN. In this study, new molecules were found be aberrantly expressed in osteosarcoma, which suggested the potential value of PODN.

The shortage of this study was that we only carried out bioinformatics analysis, without sufficient laboratory data to verify and unclear of specific molecular transmission signaling pathway. However, ACTA2, COL6A1, FAP, OLFML2B and COL6A3, these five potential targets have great research value for further study. Disclosure of these five crucial genes will greatly save the time and economic costs of osteosarcoma scholars and scientists for other researchers around the world. Our current research was just an introduction, under the guidance of our research data, we hope to see the publication of more and more valuable researches, and human beings will continue to advance their understanding and treatment of osteosarcoma.

## Conclusions

It was identified that PODN was a prognostic biomarker and correlated with immune infiltrates in osteosarcoma, and was correlated with immune infiltrates in osteosarcoma. Furthermore, ACTA2, COL6A1, FAP, OLFML2B and COL6A3, can be used as prognosis biomarkers for osteosarcoma.

## Data Availability

https://www.ncbi.nlm.nih.gov/geo/query/acc.cgi?acc=GSE42352. https://ocg.cancer.gov/programs/target/data-matrix.
